# Occupational radiation dose to the lens of the eye of medical staff who assist in diagnostic CT scans

**DOI:** 10.1016/j.heliyon.2021.e06063

**Published:** 2021-01-30

**Authors:** Keisuke Nagamoto, Takashi Moritake, Koichi Nakagami, Koichi Morota, Satoru Matsuzaki, Shun-ichi Nihei, Masayuki Kamochi, Naoki Kunugita

**Affiliations:** a Department of Radiology, Hospital of the University of Occupational and Environmental Health, Japan, 1-1 Iseigaoka, Yahatanishi-ku, Kitakyushu, Fukuoka, Japan; b Department of Radiobiology and Hygiene Management, Institute of Industrial Ecological Sciences, University of Occupational and Environmental Health, Japan, 1-1 Iseigaoka, Yahatanishi-ku, Kitakyushu, Fukuoka, Japan; c Department of Radiology, Shinkomonji Hospital, 2-5 Dairishinmachi, Moji-ku, Kitakyushu, Fukuoka, Japan; d Intensive Care Unit, Hospital of the University of Occupational and Environmental Health, Japan, 1-1 Iseigaoka, Yahatanishi-ku, Kitakyushu, Fukuoka, Japan; e Department of Occupational and Community Health Nursing, School of Health Sciences, University of Occupational and Environmental Health, Japan, 1-1 Iseigaoka, Yahatanishi-ku, Kitakyushu, Fukuoka, Japan

**Keywords:** Occupational dose to the lens of the eye, CT-assisting personnel for diagnostic CT scan, Radio-photoluminescent glass dosimeter, Multiple protective measures, Industrial health management

## Abstract

**Purpose:**

We investigated occupational dose to the lens of the eye for CT-assisting personnel for diagnostic purposes using a radio-photoluminescent glass dosimeter (RPLD) and evaluate compliance with the new equivalent dose limit for the lens of the eye (20 mSv/year). Further, we proposed the implementation of “multiple protective measures” and estimated its effect.

**Method:**

An eye lens dosimeter clip was developed specifically to attach RPLDs inside radiation safety glasses in an L-shape. Using a total of six RPLDs attached to the radiation safety glasses, the 3-mm dose-equivalent (H_p_(3)) to the lens of the eye for medical staff (n = 11; 6 intensive care physicians, 2 pediatricians, 3 radiological technologists) who assisted patients during CT scan for “diagnostic” purpose (n = 91) was measured. We evaluated the dose reduction efficiencies with radiation safety glasses and bag-valve-mask extension tube. We also estimated the protection efficiency with radiation protection curtain introduced in front of the staff's face via the phantom experiment.

**Results:**

Without wearing radiation safety glasses, H_p_(3) to the lens of the eye was greatest for intensive care physicians (0.49 mSv/procedure; allowing 40 procedures to be performed annually), followed by pediatricians (0.30 mSv/procedure; 66 procedures annually) and radiological technologists (0.28 mSv/procedure; 71 procedures annually). Use of each type of protective tools: radiation safety glasses (0.07-mm-Pb), bag-valve-mask extension tube (20 cm) and radiation protective curtain (0.25-mm-Pb), reduced H_p_(3) to the lens of the eye by 51%, 31% and 61%, respectively.

**Conclusion:**

Intensive care physicians perform most assisted ventilations with the bag-valve-mask during “diagnostic” CT scans, and may exceed the equivalent dose limit for the lens of the eye if radiation safety glasses are not worn. If “multiple protective measures” are implemented, compliance with the equivalent dose limit for the lens of the eye should be achievable without placing significant burdens on physicians or medical institutions.

## 1 Introduction

The threshold dose for radiation-induced cataract has been thought to be 5 Gy for acute exposure and 8 Gy for hyperfractionated or prolonged exposure [1]. However, numerous epidemiological studies have shown that the threshold dose may significantly decrease [2, 3, 4]. The International Commission on Radiological Protection (ICRP) in 2011 in the Seoul Statement therefore lowered the threshold dose of cataract to 0.5 Gy and recommended a new equivalent dose limit for the lens of the eye, to specify occupational exposure in planned exposure situations, “For occupational exposure in planned exposure situations the Commission now recommends an equivalent dose limit for the lens of the eye of 20 mSv in a year, averaged over defined periods of 5 years, with no single year exceeding 50 mSv” [5]. Since this recommendation, increased interest has been shown in cataracts and occupational dose to the lens of the eye for medical staff in Japan.

Many reports have shown that occupational dose to the lens of the eye is significant among physicians involved in clinical radiation care over many hours, such as interventional radiology (IR) of the neurovascular system [6, 7, 8, 9], cardiovascular system [8, 10] or tumor [8, 9, 11], and endoscopic retrograde cholangiopancreatography (ERCP) [8, 12, 13, 14]. The risk of radiation-induced cataracts is widely recognized. However, relatively little is known about occupational doses to the lens of the eye in CT-assisting personnel. CT has become commonly used in recent years for “treatment” purposes, such as tissue biopsy and drainage. Several reports have examined occupational dose to the lens of the eye in physicians performing these procedures [15, 16, 17, 18, 19, 20, 21, 22, 23] (Table 1), and special protective equipment and their effects in dose reduction [22, 24, 25, 26]. However, while reports have already investigated nurses assisting pediatric patients in the CT room during CT scans for “diagnostic” purposes [27] or in phantom experiments [28, 29], no reports have described direct measurement in medical staff assisting adult patients undergoing high-dose imaging procedures.

**Table 1:** Occupational dose to the lens of the eye during CT fluoroscopy for “treatment” purposes: literature review.

Author (year of publication) [reference number]	Measurement target	Type of dosimeter	Measurement position	Dose
Silverman et al. (1999) [15]	Phantom	Ionization chamber	Neck	2.6 μC/kg (10 mR) (scattered X-ray 100 cm from the beam)
Daly et al. (1999) [16]	Operator	Film badge	Outside protective apron (neck)	0.1–0.3 mSv/month
Mellenberg et al. (1999) [17]	Phantom	Ionization chamber	Outside protective apron (collar)	0.24 ± 0.14 mR/sec
			Outside protective apron (body)	1.13 ± 0.49 mR/sec
Paulson et al. (2001) [18]	Operator	OSLD	Outside protective apron (Lens of the eye)	0.007–0.048 mSv/procedure
Joemai et al. (2009) [19]	Operator	EPD Mk II	Outside protective apron	IR: 14 μSv/procedure
				AR: 5 μSv/procedure
				RT: 1 μSv/procedure
Heusch et al. (2014) [20]	Operator	EDD-30	Side of protective glasses	3.3 μSv/procedure (range, 0.03–218.9 μSv/procedure)
Gyekye et al. (2016) [21]	Phantom	MCNPX	Eye	4.5 ± 1.3 μGy/procedure (with protective equipment)
				4.8 ± 1.3 μGy/procedure (without protective equipment)
Sarmento et al. (2018) [22]	Operator	TLD	Outside protective apron (body)	0.06 mSv/procedure
Pravata et al. (2018) [23]	Operator	EDD-30	Outside protective apron (body)	8.2 μGy/procedure (DLP: 124 mGy･cm)

OSLD, optically stimulated luminescence dosimeter.

EPD MkII, electronic personal dosimeter (Siemens Environmental Systems, Munich, Germany).

EDD-30, educational direct dosimeter (Unfors Instruments, Billdal, Sweden).

MCNPX, Monte Carlo N-Particle eXtended.

IR, interventional radiologist.

AR, assisting radiologist.

RT, radiologic technologist.

In this study, our first objective was to develop a dosimeter that can measure the occupational dose to the lens of the eye for CT-assisting personnel in a single procedure using a radio-photoluminescent glass dosimeter (RPLD) (GD-352M; Chiyoda Technol Co., Tokyo, Japan). Our second objective was to investigate occupational dose to the lens of the eye for CT-assisting personnel during “diagnostic” CT using this dosimeter, and to evaluate compliance with the new equivalent dose limit for the lens of the eye. Finally, our third objective was to propose implementation of “multiple protective measures” that do not physically burden the CT-assisting personnel, especially intensive care physicians, and do not financially burden the medical institution through superfluous radiation protective measures, and to estimate the effects of this proposal.

## 2 Material and methods

### 2.1 Participants for occupational dose measurement

The 3-mm dose-equivalent (H_p_(3)) inside and outside the radiation safety glasses was measured for 11 medical staff as an assessment of the equivalent dose to the lens of the eye. A total of 91 “diagnostic” CT scans that needed special assistance for a patient in the CT room performed at our hospital between June 2017 and May 2018 were analyzed (Table 2).

**Table 2:** Patient information by site of CT scans for diagnostic purpose (n = 91 procedures, June 2017–May 2018).

CT imaging site	n	Age [y]	Body weight [kg]
		Mean ± SD	Mean ± SD
Head	35	41.9 ± 32.7	40.4 ± 24.7
Head, neck	3	78.3 ± 1.9	54.4 ± 1.9
Chest	5	33.6 ± 25.6	46.4 ± 22.6
Chest to abdomen	22	70.5 ± 15.1	56.5 ± 9.4
Abdomen	4	55.5 ± 33.0	55.5 ± 16.8
Head, chest	4	57.0 ± 16.5	62.2 ± 11.0
Head, Chest to abdomen	14	63.9 ± 21.1	60.5 ± 7.8
CTA	4	76.3 ± 4.5	62.7 ± 5.9

CTA, computed tomography angiography.

SD, standard deviation.

### 2.2 CT imaging method and CT dose index

Multi-slice CT systems (Aquilion ONE or Aquilion PRIME; Canon Medical Systems, Nasu, Japan) were used in this study. For image generation, auto-exposure control, and iterative reconstruction were applied. For optimization of the imaging dose, from the 1,203 CT imaging cases at our hospital seen between January 1 and June 30, 2018, median values for CTDI_vol_ and DLP per major site were individually determined and compared with the Japan Diagnostic Reference Levels for 2015 (Japan DRLs 2015) [30].

### 2.3 Method for measuring occupational dose to the lens of the eye

**Figure 1:**
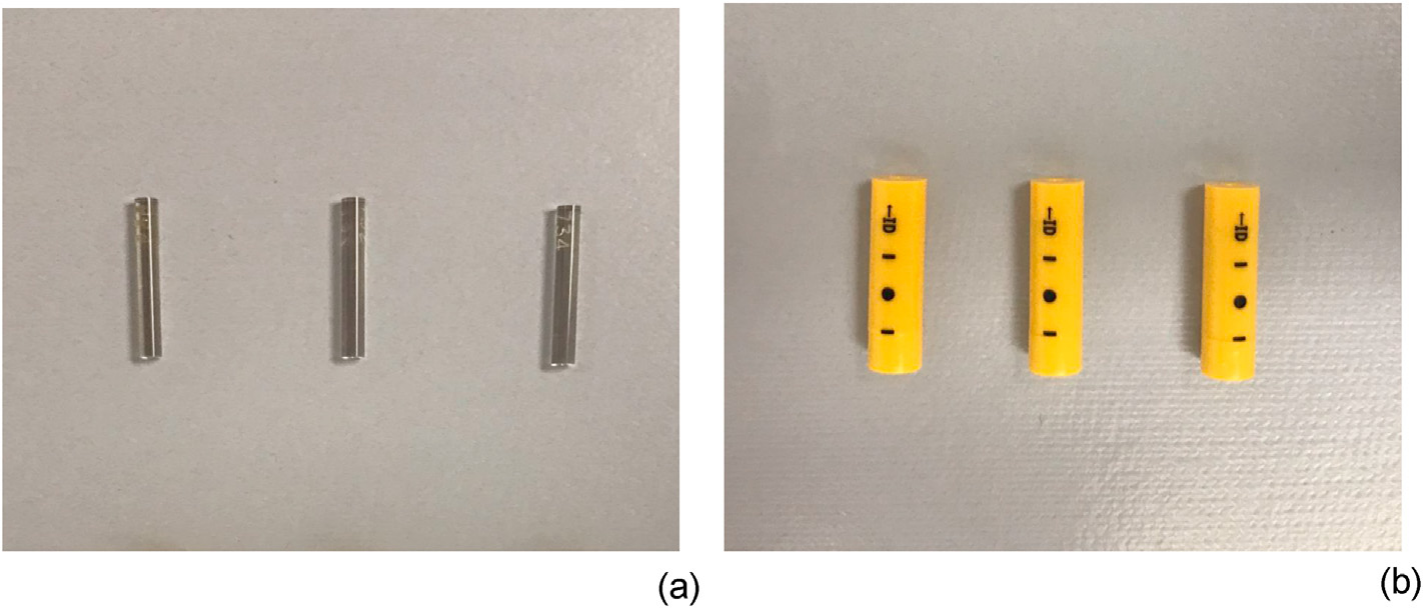
Radio-photoluminescent glass dosimeter (GD-352M). (a) The dosimeter tip is cylindrical, measuring 1.5 mm in diameter and 12.0 mm in length. (b) The tip is used in a plastic case laminated with a 0.75-mm Sn filter.

CT-assisting personnel wore radiation protective clothing (MSA-25L, 0.25-mm-Pb; Maeda Co., Tokyo, Japan) and radiation safety glasses (Panorama shield ® ultra-light 0.07-mm-Pb; Toray, Tokyo, Japan), and the H_p_(3) to the lens of the eye was obtained from the air kerma value measured by GD-352M (Figure 1) affixed inside and outside the radiation safety glasses. GD-352M complies with the IEC62387 requirements to the dosimetry system with passive detectors, offering stable dose linearity from a low dose range (not more than ± 5.0% within a range from 0.01 mGy to 50 mGy) [31, 32]. We confirmed that the coefficient of variation did not exceed 3.0% before commencing this study.

**Figure 2:**
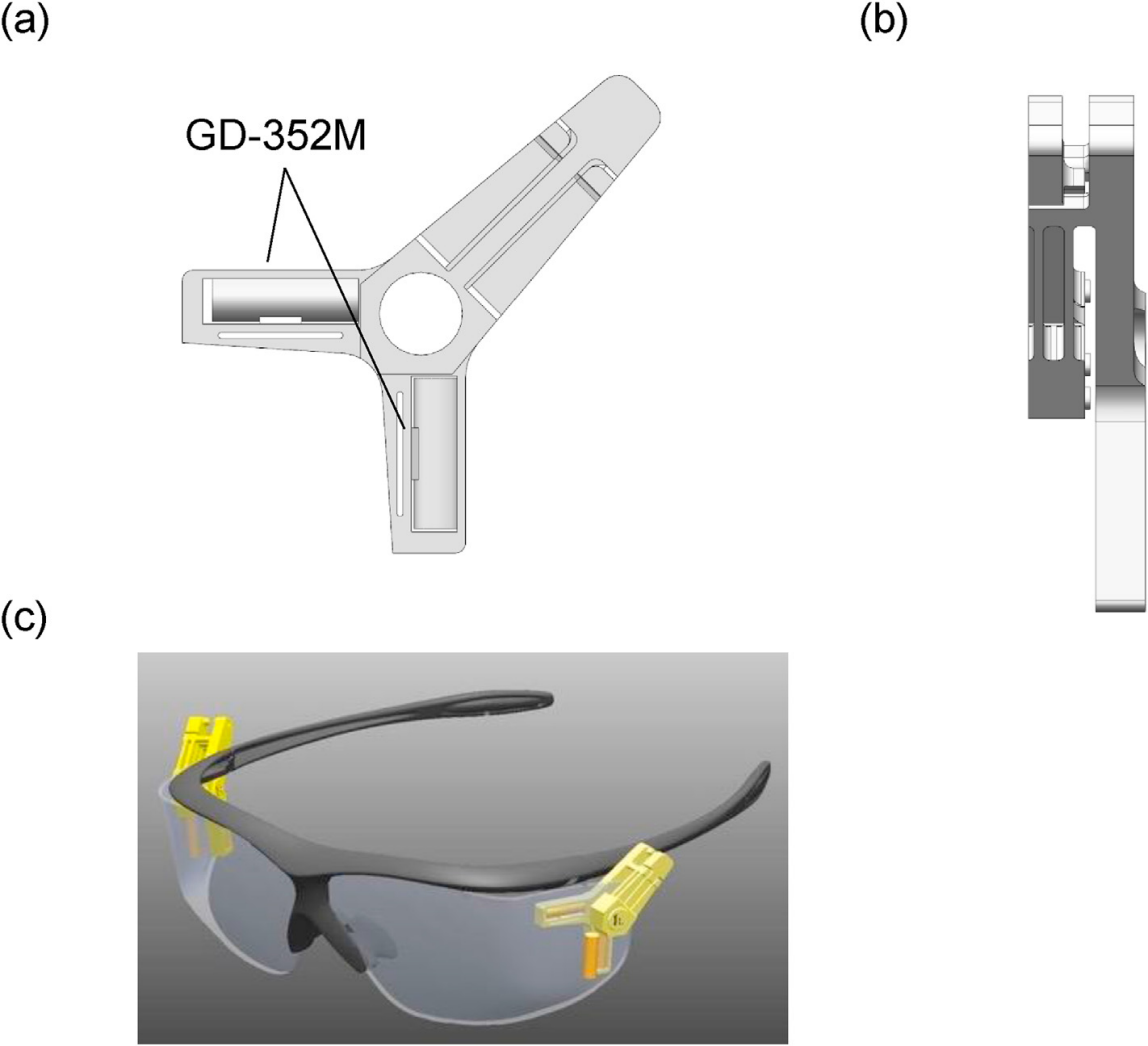
Diagram of the eye lens dosimeter clip. A polyphenylene sulfide resin clip was shaped such that the long axes of the two GD-352M were fixed perpendicular to each other inside the radiation safety glasses. (a) Anteroposterior view and (b) lateral view of the eye lens dosimeter clip. (c) Two eye lens dosimeter clips (left and right sides) were attached to the inside of the radiation safety glasses, and two GD-352M (left and right sides) were attached to the outside of the radiation safety glasses.

The GD-352M, to compensate for excessive energy response due to the high effective atomic number of the detector material, contains a Sn filter to absorb low-energy photon. However, because using this Sn filter creates a directional dependence of sensitivity [31], an eye lens dosimeter clip made with polyphenylene sulfide resin was manufactured such that two GD-352M RPLDs were placed in an L-shape, vertically and horizontally in relation to the ground, thereby offsetting the directional dependence and having the GD-352M does not hamper the user as well (Figure 2a, b). Using this eye lens dosimeter clip, a total of six GD-352M were attached to the inside and outside of the radiation safety glasses (Figure 2c).

Imaging settings of the CT (tube voltage, tube current), CT dose indices CTDI_vol_ and DLP, position of the CT-assisting personnel, method of assistance, and method of radiation protection were recorded per CT scan.

### 2.4 Conversion of Hp(3) to the lens of the eye from air kerma value obtained from RPLD

X-ray tube position of the CT scanner was fixed at 0° and tube voltage was set at 120 kVp. Using the wireless X-ray output analyzer (Piranha Model 657; RTI, Mölndal, Sweden) placed at the isocenter of the CT gantry, the aluminum half-value layer (Al-HVL) was measured. Next, the X-ray spectrum was calculated using an approximate expression by Tucker et al. [33], and the effective energy was determined from the obtained Al-HVL using the interaction cross-section data of photons and matter by Hubbell et al. [34]. Mean effective energy was set as 56.47 keV (Table 3). Finally, the “air kerma to 3-mm dose-equivalent conversion coefficient K (H_p_(3)/air kerma),” which corresponds to the mean effective energy, was taken from previous reports [35]. Conversion coefficient K was determined to be 1.650.

**Table 3:** X-ray effective energy during CT scan at a tube voltage of 120 kVp.

CT equipment	FOV	Effective energy [keV]
80-MDCT	S-size (24 cm)	55.74
	L-size (40 cm)	57.97
320-MDCT	S-size (24 cm)	54.87
	L-size (40 cm)	57.30
Mean ± SD		56.47 ± 1.23

FOV, field of view.

80-MDCT, 80 multidetector-row CT.

320-MDCT, 320 multidetector-row CT.

SD, standard deviation.

### 2.5 Analysis of the reduction in occupational dose to the lens of the eye with radiation protective equipment

**Figure 3:**
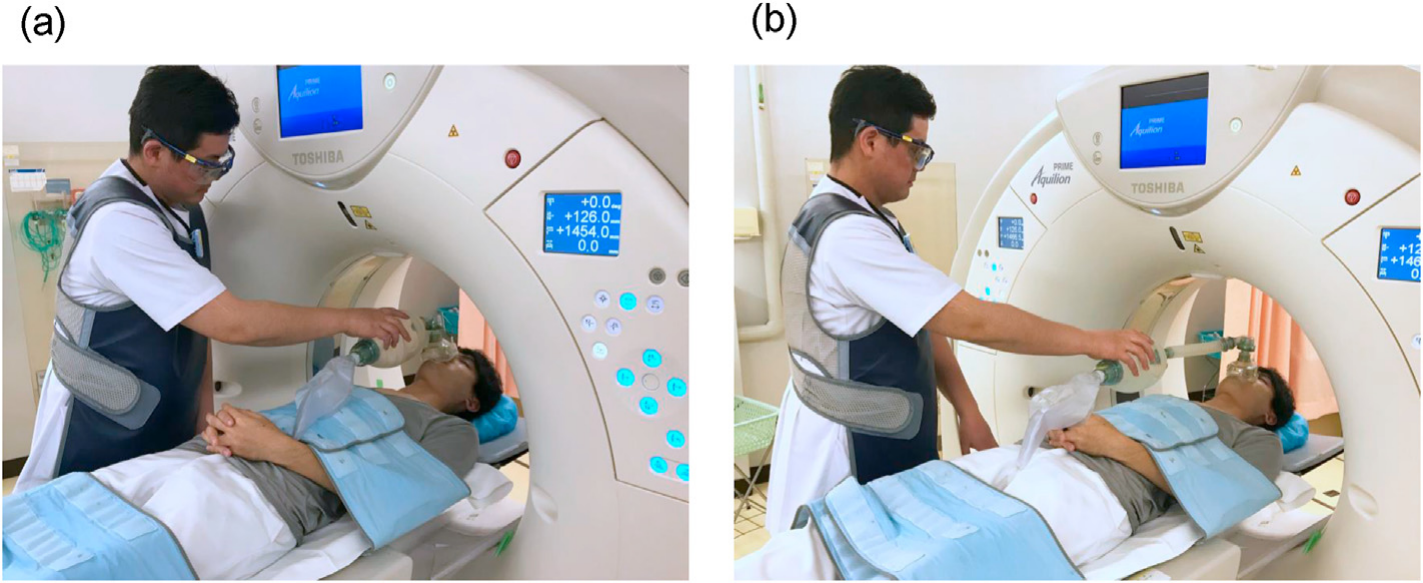
Use of a bag-valve-mask extension tube (approximately 20 cm). (a) Without extension tube. (b) With extension tube.

**Figure 4:**
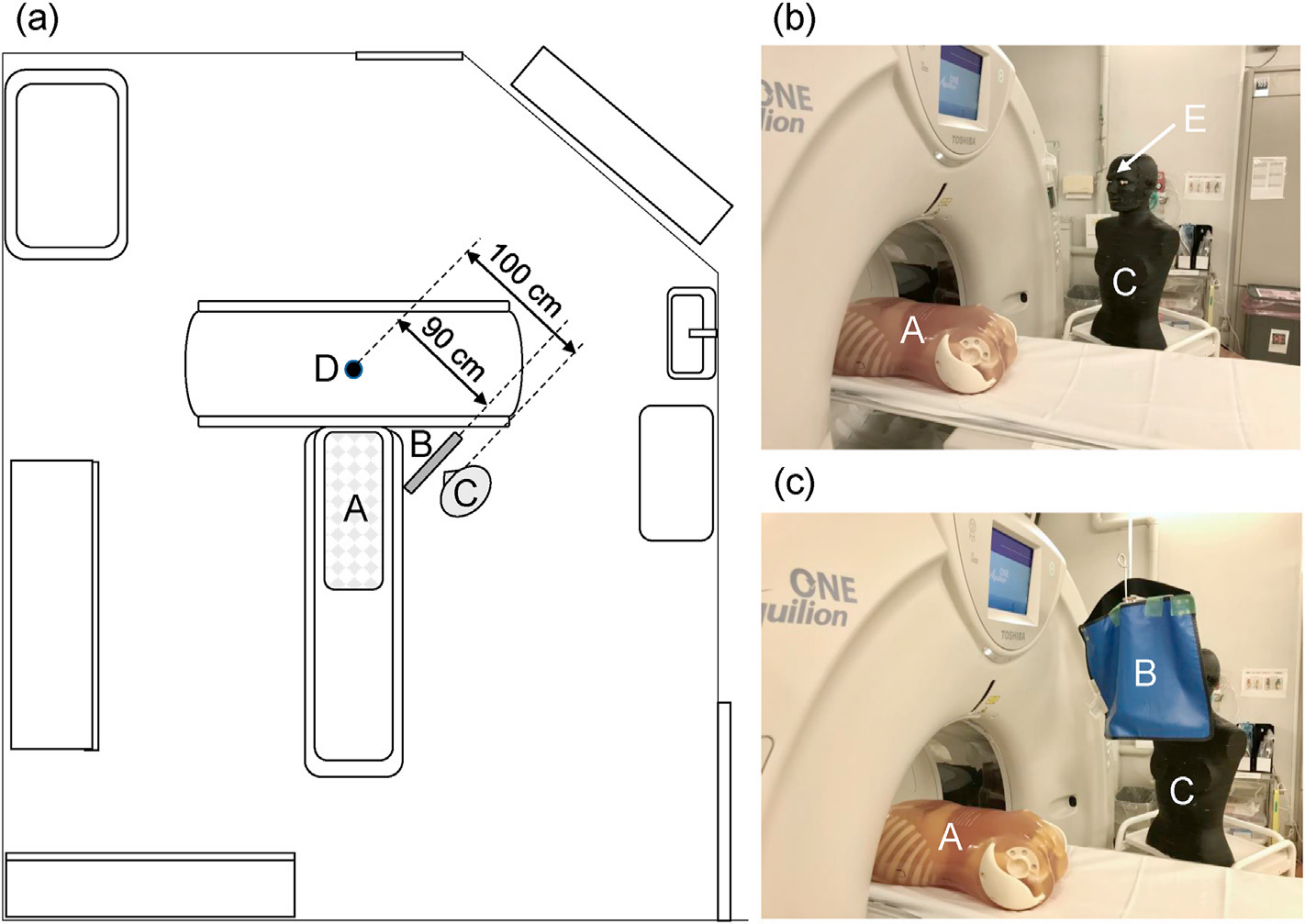
Experiment setting for obtaining dose reduction efficiency with the radiation-protective curtain. (a) Geometry arrangement for measurements. (b) Without radiation protective curtain. (c)With radiation protective curtain. A: Body phantom instead of patient (PBU-60, Kyoto Kagaku Co., Kyoto, Japan). B: Radiation protective curtain (39 × 43 cm, 0.25-mm Pb-equivalent thickness, 90 cm from the CT isocenter, with the upper edge of the curtain at a height of 185 cm from the ground). C: Body phantom instead of CT-assisting personnel (Alderson RANDO phantom). D: Isocenter of CT gantry. E: GD-352M affixed at the forehead of the phantom (100 cm from the CT isocenter, at a height of 160 cm from the ground).

From the H_p_(3) dose ratio of outside to inside the radiation safety glasses (outside/inside) measured for CT-assisting personnel, the dose reduction efficiency with radiation safety glasses was determined. After each H_p_(3) was normalized to the corresponding DLP, the dose reduction efficiency with the bag-valve-mask extension tube was determined from the median dose ratio using the bag-valve-mask extension tube compared to not using the extension tube (with/without) (Figure 3). Furthermore, using a CT scanner at 120 kVp of tube voltage, the dose reduction efficiency with the radiation protective curtain was experimentally determined. A radiation protective curtain (39 × 43 cm, 0.25-mm Pb-equivalent thickness) was installed at the position 10 cm away from the face of the phantom (Alderson RANDO phantom, Radiology Support Device Inc., California, US) and the H_p_(3) was analyzed with the GD-352M that had been affixed at the forehead of the phantom. The height of GD-352M was adjusted at 160 cm from the ground the eye level of the average Japanese male (Figure 4).

### 2.6 Statistics

Occupational doses, CT imaging settings, and CT dose indices were compared between different standing positions of the assisting personnel and between professions of the personnel using Kruskal-Wallis one-way analysis of variance (ANOVA). When statistical significance was observed with one-way ANOVA, the difference between standing positions of each CT-assisting personnel and between professions of these personnel was evaluated using Dunn's test (with Bonferroni correction). Values of P < 0.05 were considered statistically significant.

### 2.7 Ethics

This study was approved by the ethics committee at our university (approval number: R1-054) and was conducted in compliance with the tenets of the Declaration of Helsinki.

## 3 Results

### 3.1 Optimization of CT settings

Comparing the CT dose index at our hospital with the Japan DRLs 2015, nearly all CT dose indices (median) were lower than the Japan DRLs 2015, demonstrating sufficient optimization (Table 4).

**Table 4:** Comparison between CT dose indices at our hospital (n = 1,203 procedures, January 1− June 30, 2018) and diagnostic reference levels.

Examinations	n	Body weight [kg]	CTDIvol [mGy]				DLP [mGy·cm]			
		Mean ± SD	Mean ± SD	Median [range]	25th−75th percentiles	Japan DRLs∗	Mean ± SD	Median [range]	25th−75th percentiles	Japan DRLs∗
*Adult*										
Routine brain	219	56.1 ± 13	54.7 ± 14.4	52.8 [32.4–136.3]	47.1–58.4	85	936.3 ± 277.3	865.1 [528.7–2319.8]	775.1–1,000.4	1,350
Routine chest	294	56.6 ± 12.7	11.1 ± 4.1	10.6 [3.8–34.4]	8.4–13.2	15	414.5 ± 137.1	405.2 [138.3–1205.0]	318.4–491.6	550
Chest to pelvis	234	57.1 ± 12.6	13.4 ± 5.1	12.3 [5.7–29.5]	9.8–16.0	18	967.1 ± 389.0	894.7 [392.2–2252.2]	691.0–1,157.0	1,300
Abdomen to pelvis	82	54.9 ± 11.6	18.6 ± 8.9	17.8 [7.4–50.3]	10.4–24.5	20	898.3 ± 400.2	876.9 [349.1–1934.4]	507.3–1,196.0	1,000
Liver, multi-phase	54	59.8 ± 12.0	18.5 ± 5.1	18.4 [8.3–32.1]	15.2–21.7	15	2,254.1 ± 728.8	2,196.6 [991.5–3891.4]	1,767.2–2,507.9	1,800
Coronary CTA	50	61.5 ± 13.6	68.0 ± 33.3	64.5 [10.6–191.8]	47.2–91.4	90	1,411.4 ± 614.9	1,356.8 [400.6–4537.8]	400.6–1,691.7	1,400

*Child* (age, years)										
Head (0)	47	3.9 ± 2.4	21.6 ± 9.3	20.2 [1.3–47.0]	14.9–26.5	38	394.7 ± 198.4	379.1 [21.5–1,011.4]	246.4–464.2	500
Head (1–5)	58	12.8 ± 5.4	32.0 ± 10.8	30.1 [9.1–82.2]	24.7–40.9	47	684.7 ± 256.7	669.6 [300.6–2,316.4]	520.0–832.7	660
Head (6–10)	47	23.1 ± 7.2	40.1 ± 13.6	36.9 [20.3–95.2]	32.9–44.8	60	799.5 ± 243.3	763.5 [361.0–1,498.0]	667.2–924.8	850
Chest (0)	18	3.5 ± 0.9	6.5 ± 2.9	6.5 [2.2–11.1]	4.6–8.5	11	121.6 ± 79.5	110.6 [26.9–348.8]	57.8–151.6	210
Chest (1–5)	25	13.2 ± 3.8	7.9 ± 5.3	5.1 [2.7–23.0]	4.6–9.5	14	209.7 ± 151.6	174.2 [68.6–646.2]	82.4–230.7	300
Chest (6–10)	19	19.1 ± 5.2	9.7 ± 5.5	8.8 [3.3–21.5]	5.1–12.8	15	275.0 ± 159.3	212.7 [86.8–629.8]	136.5–408.6	410
Abdomen (0)	17	5.1 ± 2.7	5.7 ± 3.1	5.3 [1.1–11.1]	4.2–8.4	11	137.3 ± 87.8	104.1 [23.5–300.0]	62.2–207.0	220
Abdomen (1–5)	19	13.8 ± 2.4	9.8 ± 3.7	9.5 [4.2–17.9]	8.1–10.8	16	313.5 ± 122.7	311.7 [96.0–602.7]	217.1–363.9	400
Abdomen (6–10)	20	22.7 ± 5.3	8.8 ± 4.6	6.7 [4.1–19.0]	5.5–14.0	17	337.5 ± 154.7	250.1 [166.5–611.4]	237.4–550.3	530

CTDIvol, computed tomography dose index (volume).

CTA, computed tomography angiography.

DLP, dose-length product.

DRLs, diagnostic reference levels.

∗ Japan Diagnostic Reference Levels 2015 (Japan DRLs 2015).

### 3.2 Directional dependence of GD-352M in radiation sensitivity

Analysis of the dose ratio (vertical/horizontal) of the two GD-352M positioned orthogonally in vertical and horizontal directions relative to the ground showed that the vertical-to-horizontal dose ratio was virtually 1.0 for both left and right sides (Table 5). As the directional dependence of the GD-352M sensitivity was found to be practically negligible, the dose value of the vertical or horizontal GD-352M that provided the higher dose value was used.

**Table 5:** Vertical/horizontal dose ratio of GD-352M inside the radiation safety glasses (n = 91 procedures, June 2017–May 2018).

Side of radiation safety glasses	Vertical-to-horizontal dose ratio (V/H)∗		
	Mean ± SD	Median [range]	95%CI
Right (n = 91)	1.03 ± 0.03	1.00 [0.72–3.33]	0.97–1.08
Left (n = 91)	1.00 ± 0.02	0.98 [0.49–1.78]	0.97–1.04

SD, standard deviation.

95%CI, 95% confidence interval of mean value.

∗ Vertical-to-horizontal dose ratio of GD-352M placed perpendicular to the ground (vertically) and parallel to the ground (horizontally) inside the radiation safety glasses.

### 3.3 Bilateral difference in occupational dose to the lens of the eye

Bilateral dose ratios (right/left) for both outside and inside the radiation safety glasses were approximately 1.0, indicating an absence of clear bilateral differences (Table 6). The greater dose value from either the right or left side was used as the H_p_(3) to the lens of the eye.

**Table 6:** Bilateral dose ratio of GD-352M (n = 91 procedures, June 2017–May 2018).

Outside/Inside radiation safety glasses	Bilateral dose ratio (right/left)∗		
	Mean ± SD	Median [range]	95%CI
Outside (n = 91)	1.04 ± 0.61	0.95 [0.17–4.81]	0.91–1.16
Inside (n = 91)	1.02 ± 0.23	0.99 [0.49–1.78]	0.97–1.07

95%CI, 95% confidence interval of mean value.

SD, standard deviation.

∗ Dose ratio of GD-352M placed on the right and left sides of the radiation safety glasses.

### 3.4 Working practice and occupational dose to the lens of the eye for CT-assisting personnel

**Figure 5:**
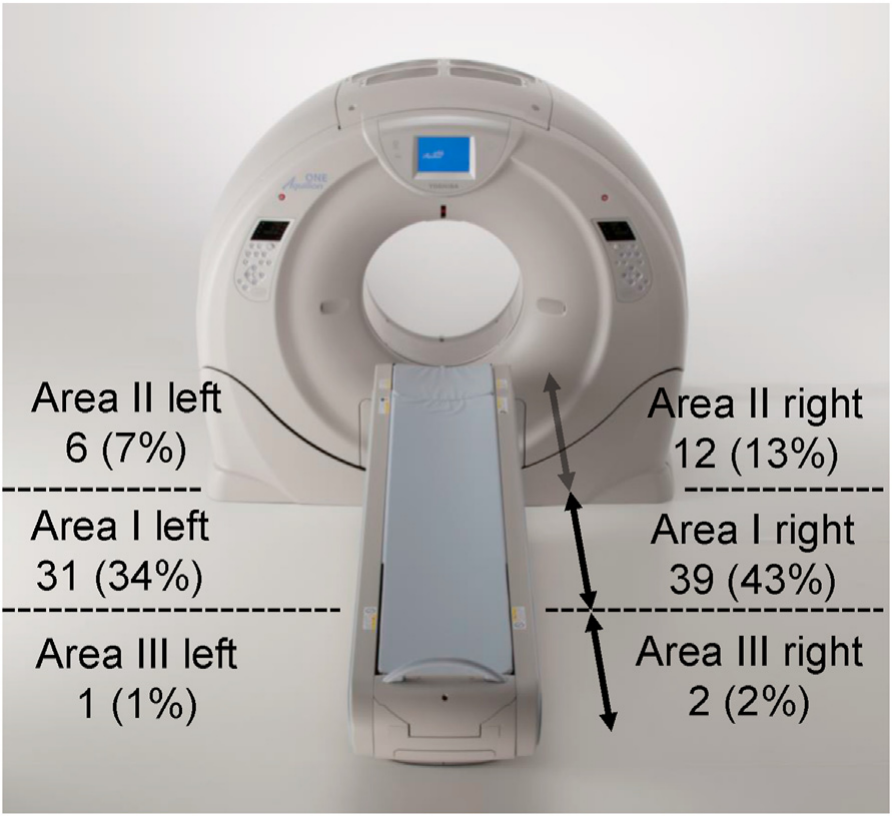
Standing positions of CT-assisting personnel and number of assists (n = 91, June 2017–May 2018). Area I is on the side of the bed near the CT gantry. Area II is on the other side of the bed and near the CT gantry. Area III is on the side of the bed away from the CT gantry. The number of personnel (number of times, %) in each position is listed.

**Figure 6:**
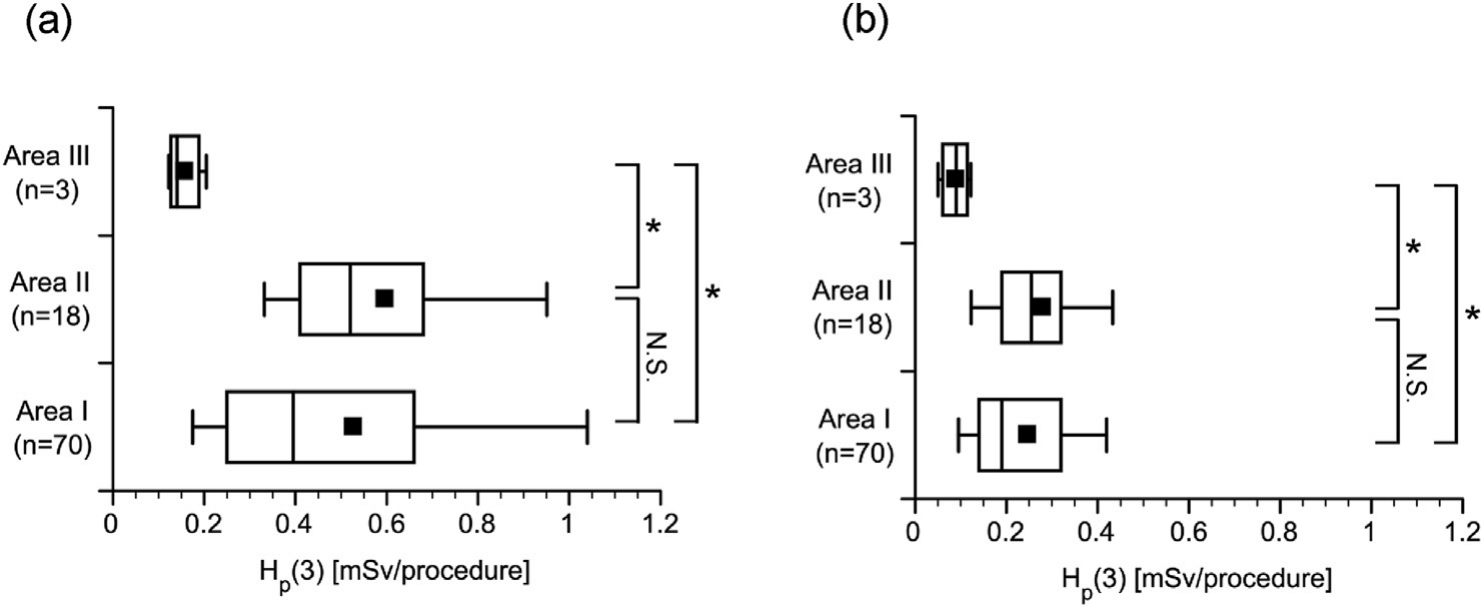
The 3-mm dose-equivalent (H_p_(3)) to the lens of the eye by standing position of the CT-assisting personnel during CT (n = 91, June 2017–May 2018). H_p_(3) to the lens of the eye of the assisting personnel per CT procedure (a) outside the radiation safety glasses and (b) inside the radiation safety glasses. ANOVA (Kruskal-Wallis) resulted in: (a) P = 0.010 and (b) P = 0.017. ∗ Pairs with P < 0.05 according to multiple comparison using Dunn's test (with Bonferroni correction) are shown. N.S., no statistical significance.

Working practices for CT-assisting personnel are shown in Table 7. Assistance was mostly performed in Area I and Area II near the CT gantry ((31 + 39 + 6 + 12)/91, 97%) (Figure 5). Working in Area I and Area II, compared with Area III (distant from the CT gantry), resulted in significantly higher H_p_(3) both outside (Figure 6a) and inside (Figure 6b) the radiation safety glasses.

**Table 7:** Working practice for CT-assisting personnel by profession (n = 91 procedures, June 2017–May 2018).

Working practice	Intensive care physician (n = 6)	Pediatrician (n = 2)	Radiological technologist (n = 3)	Total [procedure]
Assisted ventilation with bag-valve-mask	65	0	0	65
Head holding	0	5	7	12
Observation	7	1	6	14
Total [procedure]	72	6	13	91

No significant differences in H_p_(3) were seen between professions for both outside (P = 0.094, Kruskal-Wallis, Table 8) and inside (P = 0.108, Kruskal-Wallis, Table 8) the radiation safety glasses.

**Table 8:** Occupational doses to the lens of the eye, CT imaging settings, and CT dose indices by profession of CT-assisting personnel (n = 91 procedures, June 2017–May 2018).

Profession	Intensive care physician (n = 6, 72 procedures)		Pediatrician (n = 2, 6 procedures)		Radiological technologist (n = 3, 13 procedures)		ANOVA∗ (*P* value)	Total (n = 11, 91 procedures)	
	Mean ± SD	Median [range]	Mean ± SD	Median [range]	Mean ± SD	Median [range]		Mean ± SD	Median [range]
*Analysis point*									
Outside radiation safety glasses (H_p_(3)) [mSv]	0.55 ± 0.39	0.49 [0.12–2.49]	0.53 ± 0.42	0.30 [0.15–1.18]	0.41 ± 0.41	0.28 [0.07–1.64]	0.094	0.53 ± 0.39	0.43 [0.07–2.49]
Inside radiation safety glasses (H_p_(3)) [mSv]	0.26 ± 0.15	0.23 [0.05–1.05]	0.23 ± 0.15	0.14 [0.09–0.46]	0.19 ± 0.15	0.15 [0.06–0.65]	0.108	0.25 ± 0.15	0.21 [0.05–1.05]

*CT imaging setting, CT dose index*									
Tube voltage [kVp]	120 ± 3	120 [100–135]	107 ± 9	100 [100–120]	117 ± 7	120 [100–120]	<0.001	120 ± 6	120 [100–135]
Tube current [mA]	4,131.2 ± 2,522.4	3,847.5 [968.0–13,196.0]	720.8 ± 741.6	339.0 [211.3–2,148.0]	1,650.0 ± 1,253.8	1177 .0 [116.0–5,178.0]	<0.001	3,532.2 ± 2,570.1	3,373.0 [67.0–13,196.0]
CTDIvol [mGy]	44.5 ± 24.7	48.6 [8.8–119.9]	23.6 ± 14.4	21.0 [2.2–42.5]	51.8 ± 25.4	45.3 [10.9–100.0]	0.650	41.5 ± 25.0	44.0 [2.2–119.9]
DLP [mGy･cm]	1,802.2 ± 947.7	1,561.0 [280.4–4,824.6]	474.2 ± 367.8	360.1 [26.9–1,063.4]	1,072.0 ± 703.1	862.9 [18.1–2,543.8]	<0.001	1,577.7 ± 972.5	1,462.8 [18.1–4,824.6]

CTDIvol, volume computed tomography dose index.

DLP, dose-length product.

H_p_(3), 3-mm dose-equivalent (personal dose equivalent).

SD, standard deviation.

∗ Homogeneity of the three profession groups was confirmed using the Kruskal-Wallis test.

Considering these results together, the distance from the CT gantry obviously exerts a stronger influence on H_p_(3) than mere differences in professions. Actually, both the intensive care physicians and the pediatricians that frequently assist patients (assisted ventilation with bag-valve mask and head holding are always performed in Area I or II) near the CT gantry (Table 7) showed a higher H_p_(3) tendency than radiological technologists (Table 8), who often perform observations while away from the CT gantry (Table 7).

### 3.5 Maximum number of procedures before reaching the annual dose limit

Considering that 20 mSv is the annual dose limit for the lens of the eye, the number of procedures that can be performed on an annual basis before reaching the annual dose limit when radiation safety glasses are not worn (calculated from outside the radiation safety glasses) and worn (calculated from inside the radiation safety glasses) was shown in Table 9. Assisted ventilation with the bag-valve-mask is the most frequently performed procedure (65/91, 71%), and is entirely performed by the intensive care physician (65/65, 100%) (Table 7). If one intensive care physician provided all the assistance without wearing radiation safety glasses, the annual limit could easily be exceeded. Warning individuals to wear radiation safety glasses is clinically important to avoid exceeding the annual dose limit for the lens of the eye.

**Table 9:** Maximum number of procedures before reaching the annual dose limit (n = 91 procedures, June 2017–May 2018).

Practice	Assisted ventilation with bag-valve-mask (n = 65 procedures)		Head holding (n = 12 procedures)		Observation (n = 14 procedures)	
	(H_p_(3)) Median [range] [mSv]	Max number	(H_p_(3)) Median [range] [mSv]	Max number	(H_p_(3)) Median [range] [mSv]	Max number
*Analysis point*						
Outside radiation safety glasses	0.51 [0.14–2.49]	39∗	0.36 [0.20–1.64]	55∗	0.19 [0.07–1.46]	105∗
Inside radiation safety glasses	0.25 [0.08–1.05]	80∗	0.16 [0.12–0.65]	125∗	0.12 [0.05–0.50]	166∗

H_p_(3), 3-mm dose-equivalent (personal dose equivalent).

∗ Maximum number not exceeding 20 mSv/year, calculated by 20 [mSv]/Median [mSv] (rounded down to nearest integer).

### 3.6 Dose reduction efficiency with radiation protective equipment

Radiation safety glasses reduced the H_p_(3) to the lens of the eye by 51% (Table 10). Moreover, use of a bag-valve-mask extension tube reduced the doses outside and inside the radiation safety glasses by 43% and 31%, respectively (Table 11). Furthermore, a 0.25-mm Pb-equivalent thickness radiation protective curtain reduced the H_p_(3) by 61% (Table 12).

**Table 10:** Dose reduction efficiency with radiation safety glasses (n = 91 procedures, June 2017–May 2018).

	Mean ± SD	Median [range]	95%CI	Dose reduction rate [%] ∗∗
H_p_(3) ratio outside/inside the radiation safety glasses (outside/inside)∗	2.06 ± 0.44	2.04 [1.07–3.73]	1.96–2.15	51

SD, standard deviation; 95%CI, 95% confidence interval.

∗ Ratio of H_p_(3) outside the radiation safety glasses (higher value of either left or right) and H_p_(3) inside the radiation safety glasses (higher value of either left or right).

∗∗ (2.04 – 1)/2.04 × 100 [%].

**Table 11:** Dose reduction efficiency with bag-valve-mask extension tube (n = 65 procedures).

Practice	Without extension tube (n = 3 procedures)		With extension tube (n = 62 procedures)		Dose reduction rate∗ [%]
	H_p_(3)	H_p_(3)/DLP	H_p_(3)	H_p_(3)/DLP	
	Median [range] [mSv]	Median [range] (a) [mSv·mGy^−1^·cm^−1^ ·10^−4^]	Median [range] [mSv]	Median [range] (b) [mSv·mGy^−1^·cm^−1^·10^−4^]	
*Analysis point*					
Outside radiation safety glasses	1.79 [0.69–2.49]	5.16 [3.59–11.8]	0.50 [0.14–1.13]	2.92 [0.88–7.62]	43
Inside radiation safety glasses	0.56 [0.26–1.05]	2.18 [1.37–3.69]	0.23 [0.08–0.56]	1.51 [0.55–3.24]	31

H_p_(3), 3-mm dose-equivalent (personal dose equivalent).

DLP, dose-length product.

∗ Dose reduction rate estimated as: {1 - (b)/(a)} × 100 [%].

**Table 12:** Dose reduction efficiency with radiation protective curtain (n = 5).

Practice	None (n = 5)	Radiation protective curtain (0.25-mm-Pb)∗ (n = 5)	Dose reduction rate∗∗∗ [%]
	H_p_ (3) (a)	H_p_ (3) (b)	
	Mean ± SD [mSv]	Mean ± SD [mSv]	
*Analysis point*			
Eye level of the CT assisting personnel∗∗	0.31 ± 0.04	0.12 ± 0.03	61

H_p_(3), 3-mm dose-equivalent (personal dose equivalent).

SD, standard deviation.

∗ 39 × 43 cm, installed at 90 cm from isocenter of the CT gantry, upper edge at a height of 185 cm from the ground.

∗∗ Forehead of the Alderson RANDO phantom, 100 cm from isocenter of the CT gantry, at a height of 160 cm from the ground.

∗∗∗ Dose reduction rate estimated as: {1 - (b)/(a)} × 100 [%].

### 3.7 Case illustration: a case of high occupational dose to the lens of the eye from a single instance of assisting in CT scans

Assistance in CT imaging was performed for an 80-year-old man who developed a complication of retroperitoneal abscess after surgery for left renal pelvis cancer. Since bleeding continued, plain CT and multiphase contrast-enhanced CT were conducted to determine the cause. An intensive care physician performed assisted ventilation using a bag-valve-mask without an extension tube and worked in Area I right near the CT gantry (Figure 5) during the scan. In this case, multiple CT scans were required, resulting in a DLP of 4,824.6 mGy⋅cm, which was higher than usual (Table 8). H_p_(3) to the lens of the eye for the CT-assisting personnel was therefore extremely high, at 2.49 mSv and 1.05 mSv outside and inside the radiation safety glasses, respectively.

## 4 Discussion

The personal dose equivalent to the lens of the eye of medical staff has traditionally been monitored by a personal dosimeter installed outside the lead apron. However, after the ICRP issued a recommendation to reduce the annual equivalent dose limit for the lens of the eye from 150 mSv to 20 mSv in 2011 [5], the need to manage the dose more strictly than ever has increased, and it has become necessary to measure the dose near the eyes, considering the shielding efficiency obtained using radiation safety glasses.

However, in Japan, the only two dosimeters that can currently be used are DOSIRIS™ (Institute for Radiological Protection and Nuclear Safety: IRSN, France) and Vision™ (Landauer, Glenwood, IL). Typically, these dosimeters measure cumulative dose in 1 month and do not offer sufficient sensitivity to evaluate dose per procedure when assisting patients in a CT scan. For this reason, we constructed an eye lens dosimeter clip and applied the GD-352M dosimeter that complies with the IEC62387 requirements [32] with sufficient sensitivity and accuracy. In this study, CT-assisting personnel wearing radiation safety glasses placed two GD-352M dosimeters in an L shape under the radiation safety glasses. As a result, it was clarified that the radiation sensitivity of GD-352M was not affected by the direction. This was probably because the directions of the primarily scattered x-rays were different from the two directions of GD-352M, that is, the direction vertically or horizontally to the ground. It was also found that the field of view was not obstructed. In addition, H_p_(3) considering the shielding effect of radiation safety glasses could be obtained for each case of assisting patients. Therefore, in order to measure H_p_ (3) in single case of assistance, it would be sufficient to install one GD-352M vertically or horizontally to the ground inside the radiation safety glasses.

Our results proved that intensive care physicians, who are frequently exposed to radiation during CT for “diagnostic” purposes, had an H_p_(3) of 0.49 mSv/procedure (median) without radiation safety glasses (Table 8). Our data was about the same as the value of H_p_(3) previously reported for the CT-assistance personnel during “diagnostic” CT scans [28, 29]. Furthermore, our data showed a significantly greater H_p_(3) than the previously reported doses to the lens of the eye for the operator during CT fluoroscopy for “treatment” purposes (Table 1). Pravata *et al.* reported that the DLP value was 124 mGy･cm for “treatment” CT scans [23], whereas standard setting was about several hundred ~ mGy･cm for “diagnostic” CT scans for adult patients according to our hospital's data (Table 4). CT for “diagnostic” purposes requires a larger number of photons to be injected into the subject than CT fluoroscopy for “treatment” purposes, given the need to obtain high-resolution imaging. This was considered one of the reasons for the higher occupational dose compared to other CT-assisting personnel. Intensive care physicians thus require appropriate training on protective measures suitable to the high occupational dose to the lens of the eye.

In this study, radiation safety glasses showed a 51% dose reduction, which was almost the same as previously reported for the protective glasses with 0.07-mm Pb-equivalent thickness (51.3%–60.0%) [7, 10, 13, 28, 36, 37, 38]. Increasing the lead content of radiation safety glasses (e.g. 0.5 or 0.75-mm Pb-equivalent) can improve the shielding effect, but it will be heavier. It is important to note that if radiation safety glasses are no longer worn simply because they are heavy, they will result in no contribution to radiation protection. When analyzing dose reduction efficiency using dosimeters installed outside and inside the radiation safety glasses, a high level of uncertainty remains, due to the radiation scattered from the glasses themselves [36] and the head of the personnel as well, posture of medical staff, position relative to the X-ray source, and the equipment model [37, 38, 39]. Although uncertainties in dose reduction efficiency cannot be entirely ignored, it would be preferable for medical staff who are likely to exceed the annual dose limit for the lens of the eye to monitor inside the radiation safety glasses (when used) rather than outside the lead apron.

When considering the radiation protection of CT-assisting personnel, the three areas of the fundamentals of industrial health management [40] are important (Table 13). Following these fundamentals, if “multiple protective measures” can be implemented with the combination of bag-valve-mask extension tube and radiation safety glasses, reducing H_p_(3) to the lens of the eye by 66% (calculated by a simple multiplication, (1 – 0.31) × (1 – 0.51) × 100 = 34%) would be theoretically possible without placing any significant burden on either medical staff or the medical institution.

**Table 13:** Three areas of industrial health management for CT-assisting personnel.

Three areas of industrial health management	Specific measures
I Working environment management	Measurement of scattered radiation, radiation shielding, use of protective boards (curtain), optimization of CT imaging settings in comparison with DRLs
II Working management	Examination of CT-assistance procedures, use of personal protective equipment (radiation safety glasses, neck guards, protective clothing), use of bag-valve-mask extension tube
III Health management	Health checkup before employment (before assignment), regular health checkup (general examination; specialized tests for blood, lens of the eye, skin, etc.), health diagnosis at abnormal radiation exposure, follow-up after leaving work

DRLs, diagnostic reference levels.

Nonetheless, when an increase is seen in unforeseen cases irradiated with a high dose, as shown in the case illustration, a radiation protective curtain (Figure 4) or so (e.g. Ceiling-mounted transparent barrier) should be installed in the CT examination room. Using these three types of protection as part of the “multiple protective measures,” an 87% (calculated by a simple multiplication, (1 – 0.31) × (1 – 0.51) × (1 – 0.61) × 100 = 13%) reduction in H_p_(3) to the lens of the eye can be expected. However, installing a ceiling-mounted radiation protective curtain is not necessarily easy, due to the structural limitations of CT examination rooms, the high cost of installation, and the obstacle created to patient assistance.

## 5 Conclusions

We could measure the H_p_(3) to the lens of the eye of the CT-assisting personnel in a single procedure using GD-352M attached to the dosimeter clip developed. We also found that median H_p_(3) to the lens of the eye for intensive care physicians, who frequently perform assisted ventilation with a bag-valve-mask, was 0.49 mSv/procedure without wearing radiation safety glasses, indicating that the equivalent dose to the lens of the eye may exceed 20 mSv annually. However, following the fundamentals of industrial health, implementing “multiple protective measures” could achieve full compliance with the equivalent dose limit for the lens of the eye (average annual limit, 20 mSv/year over 5 years).

## Declarations

### Author contribution statement

T. Moritake, K. Nagamoto: Conceived and designed the experiments; Performed the experiments; Analyzed and interpreted the data; Wrote the paper.

K. Nakagami, K. Morota, S. Matsuzaki: Performed the experiments; Analyzed and interpreted the data.

S. Nihei, M. Kamochi: Performed the experiments Investigation; Contributed reagents, materials, analysis tools or data.

N. Kunugita: Contributed reagents, materials, analysis tools or data.

### Funding statement

S. Nihei was supported by JSPS KAKENHI Grant Number JP18K09959. M. Kamochi was supported by JSPS KAKENHI Grant Number JP19K10498. T. Moritake was supported by JSPS KAKENHI Grant Number JP18H03376. N. Kunugita was supported by Japanese Ministry of Health, Labour and Welfare (Grant Number: 180501-1).

### Data availability statement

Data included in article/supplementary material/referenced in article.

### Declaration of interests statement

The authors declare no conflict of interest.

### Additional information

No additional information is available for this paper.
